# Spectral Compatibility Between Photoinitiators and LED Curing Lights: Photographic and Spectroscopic Evidence from Model Resin Systems

**DOI:** 10.3390/ma19214467

**Published:** 2026-10-23

**Authors:** Afnan O. Al-Zain, Richard B. Price, Jeffrey A. Platt, Mary Anne S. Melo

**Affiliations:** 1 Restorative Dentistry Department, Faculty of Dentistry, King Abdulaziz University, P.O. Box 80209, Jeddah 21589, Saudi Arabia; 2 Department of Dental Clinical Sciences, Faculty of Dentistry, Dalhousie University, 5981 University Avenue, Halifax, NS B3H 4R2, Canada; rbprice@dal.ca; 3 Department of Biomedical and Applied Sciences, Division of Dental Biomaterials, Indiana University School of Dentistry, Indianapolis, IN 46202, USA; jplatt2@iu.edu; 4 Department of Comprehensive Dentistry, University of Maryland School of Dentistry, Baltimore, MD 21201-1786, USA; mmelo@umaryland.edu

**Keywords:** degree of conversion, spectral compatibility, light-curing unit, photoinitiator, camphorquinone, polymerization failure, esthetic restorative materials

## Abstract

**Highlights:**

- Spectral compatibility strongly influenced polymerization of unfilled model resins.
- CQ/EDAB was the only system fully polymerized with both LED units.
- TPO- and BAPO-containing resins were fully polymerized only with the multiple-emission-peak LED.
- Spectral mismatch caused incomplete polymerization and undercuring.
- Failed polymerization visualized the photoinitiator-LED spectral mismatch.

**Abstract:**

Polymerization of resin-based composites depends on an overlap between photoinitiator absorption and light-curing unit (LCU) emission, yet resins that fail to cure cannot be measured and go unreported. This study tested how photoinitiator chemistry and concentration interact with light-emitting diode (LED) emission spectrum, documenting outcomes photographically. Sixteen unfilled bisphenol A glycerolate dimethacrylate/triethylene glycol dimethacrylate (Bis-GMA/TEGDMA; 1:1 wt/wt premixed resin blend) formulations containing camphorquinone (CQ) combined with ethyl 4-(dimethylamino)benzoate (EDAB), 1-phenyl-1,2-propanedione (PPD), diphenyl(2,4,6-trimethylbenzoyl)phosphine oxide (TPO), or phenylbis(2,4,6-trimethylbenzoyl)phosphine oxide (BAPO) at four concentrations were cured for 20 s with a single-emission-peak (SLED) or multiple-emission-peak (MLED) LED unit (n = 3; 96 specimens). Specimens were photographed and classified as fully polymerized, partially polymerized, or unpolymerized; degree of conversion (DC) was measured by attenuated total reflectance Fourier-transform infrared spectroscopy (ATR-FTIR) and analyzed by two-way analysis of variance (ANOVA) and Tukey’s honestly significant difference (HSD) tests (α = 0.05). Formulation, LCU, and their interaction significantly affected DC (*p* < 0.001), and these effects remained significant when specimens assigned 0% DC were excluded. CQ+EDAB was the only system that polymerized fully with both units (58.6–76.5%). CQ+TPO achieved a maximum DC of 68.5% with the MLED, which was not statistically significantly different from the highest value obtained with CQ+EDAB. However, when polymerized with the SLED, CQ+TPO produced unpolymerized specimens that could not be measured at any of the tested concentrations. CQ+BAPO responded to both units, more strongly to the MLED (61.2% vs. 48.1%). CQ+PPD, formulated without an amine co-initiator, failed with both units (≤6.7%). Photographic classification agreed with ATR-FTIR and recorded the outcome where spectroscopy was impossible. Higher radiant exposure outside the photoinitiator absorption band did not compensate for spectral mismatch, whereas within the band, conversion also depended on irradiance and photoinitiator concentration. Spectral compatibility should therefore be verified rather than presumed.

## 1. Introduction

Successful polymerization determines nearly every clinically relevant property of a resin-based composite (RBC) restoration. Adequate monomer-to-polymer conversion supports mechanical strength, dimensional and color stability, wear resistance, and biocompatibility, whereas incomplete conversion leaves unreacted monomer within the restoration [1,2,3]. Reduced conversion has been associated with inferior mechanical performance, accelerated degradation, and elution of residual monomers with cytotoxic, genotoxic, and allergenic potential [1,2,4,5].

Camphorquinone (CQ) has remained the dominant photoinitiator in RBCs because its absorption maximum near 470 nm coincides closely with the emission of conventional blue light-emitting-diode (LED) light-curing units (LCUs), and because its Norrish Type II mechanism, excitation to a triplet state followed by electron and hydrogen transfer from a tertiary amine co-initiator, is highly efficient [2,3,6,7,8]. Its intrinsic yellow color and incomplete photobleaching, however, are increasingly difficult to reconcile with the highly translucent, bleach-shade, and single-shade materials that now define esthetic restorative practice [2,3,9]. Less chromatic alternatives have therefore been introduced. 1-Phenyl-1,2-propanedione (PPD) is also a Type II photoinitiator requiring an amine, but absorbs maximally near 410 nm [10,11]. Diphenyl(2,4,6-trimethylbenzoyl)phosphine oxide (TPO) and bisacylphosphine oxide (BAPO) are Norrish Type I initiators that cleave directly on photon absorption without a co-initiator and absorb maximally in the near-UV/violet region (≈380 nm for TPO and ≈370 nm for BAPO), with absorption extending to approximately 420 nm and 450 nm, respectively [1,2,3,9,12,13]. The move toward these materials therefore shifts the wavelengths on which clinical success depends toward the violet end of the spectrum.

Whether that light can effectively initiate polymerization depends first on spectral overlap and, within the absorption band, on the irradiance and radiant exposure delivered. The Grotthuss–Draper law establishes that only absorbed photons can drive a photochemical reaction, while the Stark–Einstein law states that each absorbed photon can excite only one molecule [14]. Together, these principles indicate that irradiance cannot compensate for inadequate spectral overlap, only photons emitted at wavelengths within the photoinitiator’s absorption spectrum can generate radicals [1,2,3,15,16,17]. Within the absorption band, the number of photons absorbed depends on the molar absorptivity of the photoinitiator at each emitted wavelength and on its concentration, and the polymerization that results depends further on the quantum yield of radical formation and on the reactivity of the radicals produced; these quantities differ substantially among dental photoinitiators [12,13,18,19]. Contemporary LED curing units are accordingly classified as single-emission-peak (SLED) devices, which emit only blue light within the CQ band, or multiple-emission-peak (MLED) devices, which add a violet emitter intended for short-wavelength initiators; the two also differ in beam homogeneity and in the spatial distribution of each wavelength across the tip, which can produce localized variation in conversion and mechanical properties [20,21,22,23]. Light transmitted through RBCs decreases with increasing increment thickness and shade, and because absorption and scattering attenuate shorter violet wavelengths more strongly than blue wavelengths, violet light reaches the deeper regions of a restoration less effectively [24,25]. Some commercial formulations combine violet-absorbing initiators with CQ specifically to broaden the usable activation window [26,27].

Most investigations of these interactions have used commercial RBCs, whose proprietary differences in resin chemistry, filler loading, pigments, optical modifiers, inhibitors, and initiator blends make the independent contribution of photoinitiator chemistry impossible to isolate [7,20,21,22,23,26,28]. Curing variables such as irradiance, exposure time, increment thickness, and tip distance have been examined extensively [29,30,31,32,33] and simplified model adhesive systems have been used in at least one study [34] but mechanistic evidence linking initiator composition alone to LED emission spectrum remains scarce. A second and less recognized gap concerns what is reported. The literature is built almost entirely on specimens that polymerize, a resin that fails to cure cannot be clamped, sectioned, or scanned, so it is excluded from analysis, recorded as missing data, or entered as a zero. The physical appearance and handling behavior of a spectrally mismatched photoinitiator–light pairing, the form in which a clinician would actually encounter the problem, have therefore never been systematically documented.

This study addressed both gaps using simplified, unfilled Bis-GMA/TEGDMA model resins, which allow photoinitiator chemistry and LED emission to vary independently of fillers, pigments, and proprietary additives. We light-cured four photoinitiator systems at four concentrations each with an SLED and an MLED unit. Critically, we photographed every specimen under standardized conditions, whether it polymerized, so we recorded polymerization outcome as a visible, physical result rather than solely as a number that a failed specimen cannot generate. This study treats polymerization failure as an outcome rather than missing data. Every specimen in a factorial photoinitiator × LED emission-spectrum design using filler-free model resins was photographed and classified by its handling behavior alongside attenuated total reflectance Fourier-transform infrared (ATR-FTIR) spectroscopy, including specimens that could not yield a spectrum. This allows the physical behavior of spectrally mismatched photoinitiator–LED combinations, as a clinician would encounter them, to be documented and related to the conversion measured in the same specimens. The alternative hypotheses were that (1) the photographically classified polymerization outcome of the unfilled model resins containing CQ+EDAB would be superior to that of the resins containing the alternative photoinitiator systems (PPD, TPO, BAPO) at the concentrations tested, with both the SLED and the MLED unit; and (2) DC would be significantly higher for the CQ+EDAB resins than for the resins containing the alternative photoinitiator systems with both LCUs, and an interaction would exist between photoinitiator system and LCU.

## 2. Materials and Methods

### 2.1. Study Design

**Figure 1:**
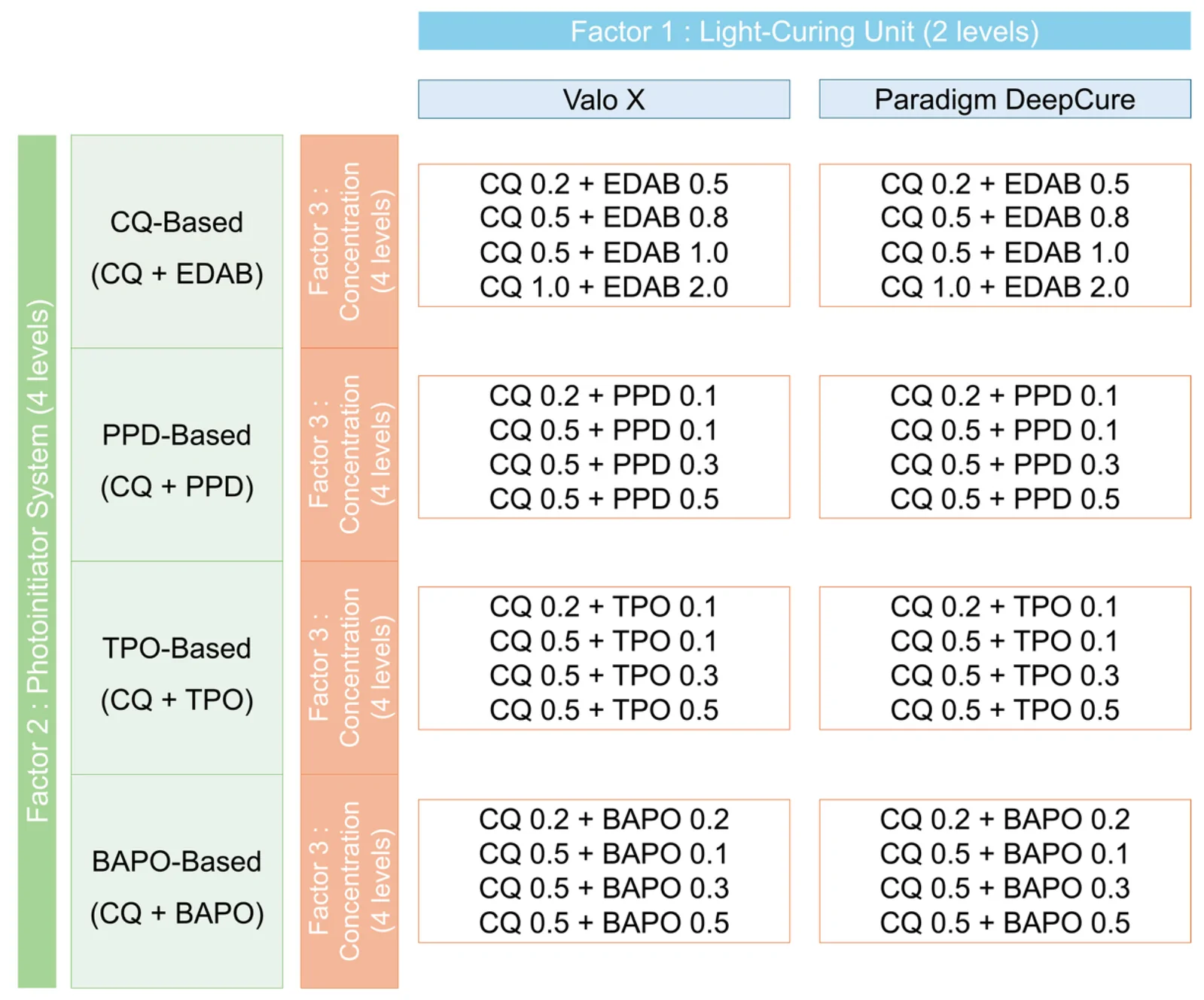
Experimental design of the study showing the photoinitiator systems, concentration ratios, and light-curing units evaluated. Abbreviations: CQ, camphorquinone; EDAB, ethyl 4-(dimethylamino)-benzoate; PPD, 1-phenyl-1,2-propanedione; TPO, diphenyl(2,4,6-trimethylbenzoyl)phosphine oxide; BAPO, phenylbis(2,4,6-trimethylbenzoyl)phosphine oxide.

This study followed a controlled laboratory experimental design to evaluate the influence of photoinitiator composition and LCU emission spectrum on resin polymerization. Four photoinitiator systems (CQ+EDAB, CQ+PPD, CQ+TPO, and CQ+BAPO) were each formulated at four photoinitiator ratios (wt%), yielding 16 distinct resin formulations. Each formulation was polymerized under two curing conditions: a SLED emitting only in the blue range and an MLED emitting both violet and blue wavelengths. This gave 32 experimental groups and enabled systematic comparison of spectral compatibility between initiator systems and curing-light emission profiles, with the conventional CQ+EDAB system serving as the spectrally matched reference against which the alternative photoinitiator systems were compared. Three specimens were prepared per formulation per LCU (n = 3), giving 96 specimens in total. Table 1 lists the materials used, and Figure 1 summarizes the experimental design.

### 2.2. Photoinitiator Characteristics

**Figure 2:**
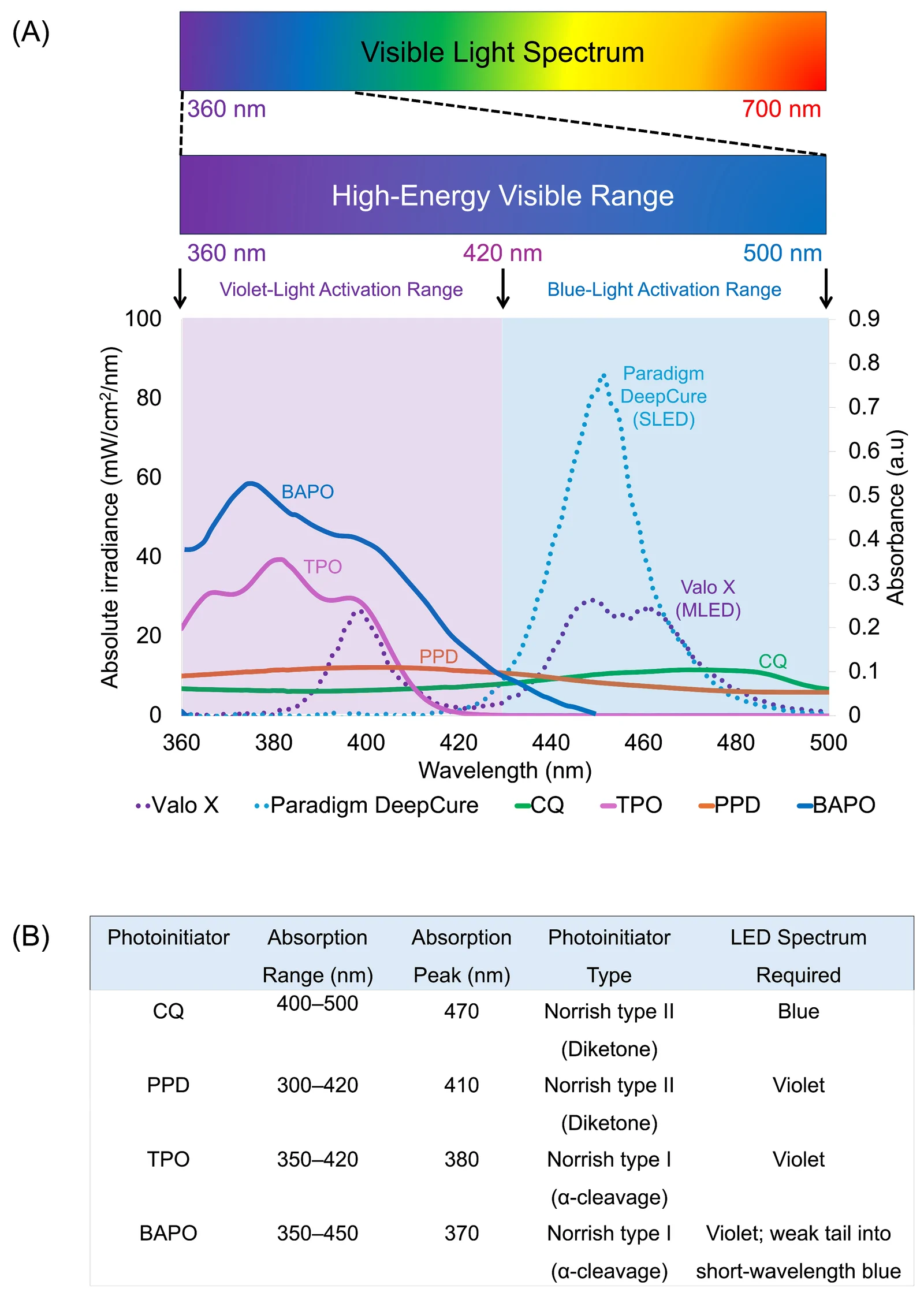
Absorption characteristics of the photoinitiators, emission ranges of the tested LED curing units, and spectral compatibility between photoinitiators and LED curing lights. (**A**) Schematic representation of the visible light spectrum and the high-energy region used for photopolymerization of dental resin materials, alongside the measured emission spectra of the two LED curing units used in this study, measured with a Managing Accurate Resin Calibrator-Patient Simulator (MARC, BlueLight Analytics Inc., Halifax, NS, Canada). The absorption spectra of CQ, PPD, and TPO were measured previously by UV-Vis spectrophotometry [3]. The BAPO spectrum is shown schematically, based on published data [13]. Absorbance is not normalized to molar concentration and therefore does not indicate relative absorption efficiency. EDAB is not plotted because it absorbs only in the ultraviolet (maximum ≈ 310 nm) and, as a co-initiator, does not need to absorb the emitted wavelength. (**B**) Summary of key characteristics of commonly used photoinitiators in dental resin systems. Abbreviations: CQ, camphorquinone; EDAB, ethyl 4-(dimethylamino)-benzoate; PPD, 1-phenyl-1,2-propanedione; TPO, diphenyl(2,4,6-trimethylbenzoyl)phosphine oxide; BAPO, phenylbis(2,4,6-trimethylbenzoyl)phosphine oxide.

Figure 2 summarizes the absorption spectra, photoinitiator classification, activation mechanisms, and corresponding wavelength requirements for each photoinitiator system. Briefly, CQ-based systems have a maximum absorption in the blue region of the visible spectrum, whereas PPD, TPO, and BAPO predominantly absorb light in the violet region. The CQ, PPD, and TPO absorption spectra in Figure 2A were measured previously with UV-Vis spectrophotometry [3] whereas the BAPO curve is a schematic representation based on published absorption data [13]. The LCU emission spectra are those measured in Section 2.3.

For each formulation, 4 g of the premixed Bis-GMA/TEGDMA (1:1 wt/wt) resin blend (weighed masses 4.006–4.120 g) was weighed into a clean, opaque container with a stir bar. The designated photoinitiator system for each experimental group was incorporated at the predetermined weight percentages expressed relative to the mass of the resin matrix; e.g., CQ 0.2 + EDAB 0.5 contained 0.008 g CQ and 0.020 g EDAB per 4 g of resin, as illustrated in Figure 1. All resin formulations (16 groups in total) were mixed on a magnetic stirrer (Variomag, Daytona Beach, FL, USA) in a room illuminated with yellow-filtered light to absorb blue wavelengths and prevent premature resin photopolymerization. Each formulation was stirred continuously in a constant-temperature room under yellow-filtered light that excludes blue light to prevent premature resin polymerization for 24 h to ensure complete dissolution of the photoinitiators and homogeneous distribution within the resin matrix.

### 2.3. Light-Curing Unit Characterization

**Figure 3:**
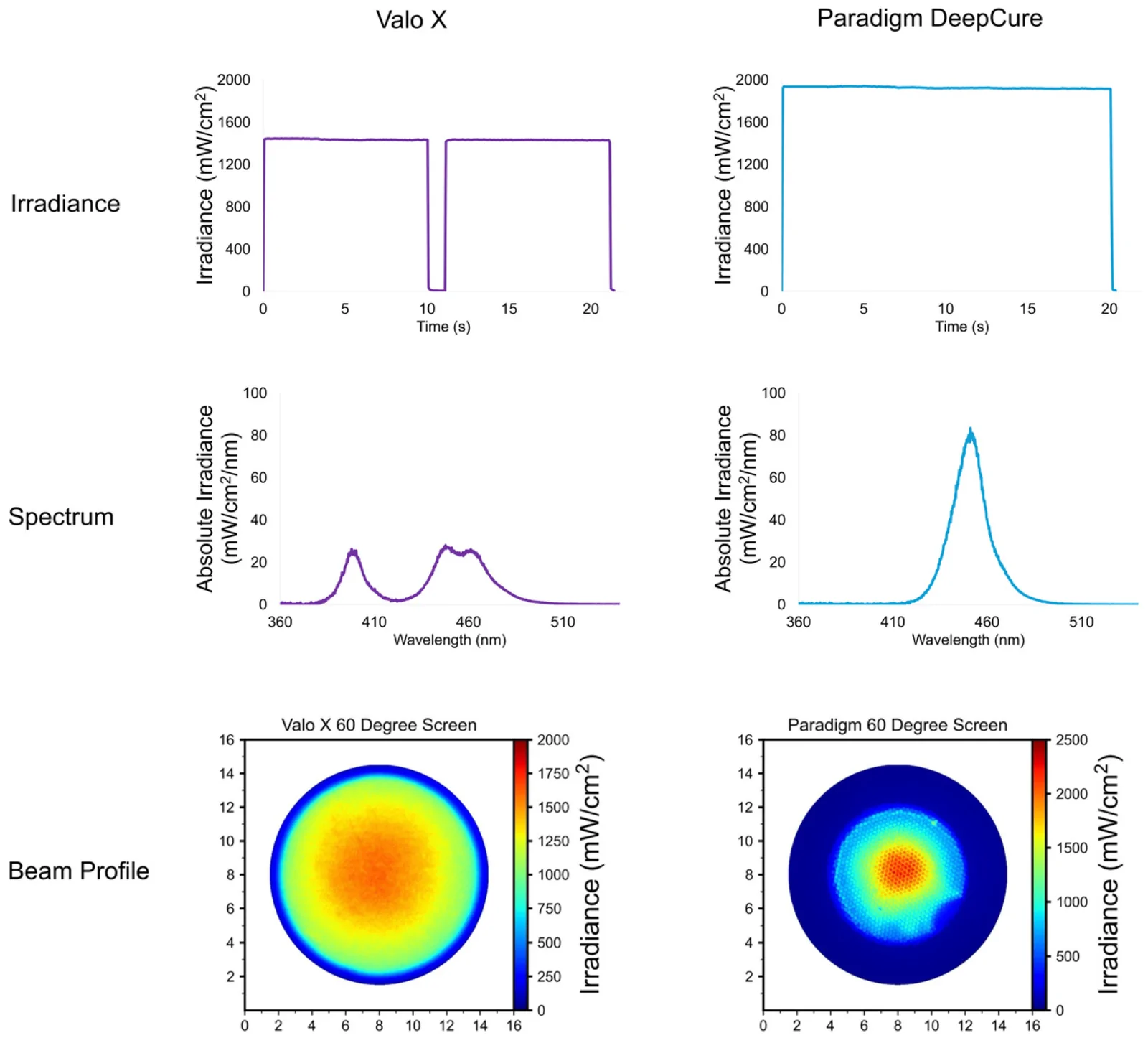
The irradiance, spectral emission, and beam profiles of the LCUs used in the study. The irradiance and spectrum of the LCUs were obtained using the anterior sensor of the calibrated Managing Accurate Resin Calibrator-Patient Simulator (MARC, BlueLight Analytics Inc., Halifax, NS, Canada). For the Valo X, the 20 s exposure was delivered as two consecutive 10 s cycles because the unit provides only 5 and 10 s exposure settings.

Two LCUs were used: an SLED unit (Paradigm DeepCure, Solventum [formerly 3M Oral Care], St. Louis, MO, USA) with a 10 mm optical tip diameter, a spectral range of 430–480 nm, an irradiance of approximately 1900 mW/cm^2^, and a radiant exposure of 38.7 J/cm^2^ over the 20 s exposure; and an MLED unit (Valo X, Ultradent Products Inc., South Jordan, UT, USA) with a 12 mm optical tip diameter, a spectral range of 385–515 nm, an irradiance of approximately 1440 mW/cm^2^, and a radiant exposure of 29.0 J/cm^2^ over the same exposure time. Because the Valo X provides only 5- and 10-s exposure settings, its 20 s exposure was delivered as two consecutive 10 s cycles, which produced the brief interruption visible in its irradiance trace (Figure 3); the Paradigm DeepCure delivered a single continuous 20 s exposure. The LCUs were fully charged before specimen preparation of each group, and light output was monitored throughout the experiment.

The irradiance, radiant exposure, and spectrum of the LCU measurements were obtained using the anterior sensor of a calibrated Managing Accurate Resin Calibrator-Patient Simulator (MARC, BlueLight Analytics Inc., Halifax, NS, Canada). Figure 3 shows the irradiance, LCU spectrum, and beam profiles of the LCUs.

### 2.4. Photographic Documentation of Polymerization Outcome

Immediately after photoactivation, we photographed specimens, including those that had not polymerized. Specimens were placed on a neutral white background under fixed, diffuse illumination and imaged with the camera mounted on a fixed stand oriented perpendicular to the specimen plane, so that working distance, viewing angle, and magnification were constant across all images. All photographs were captured on a single device (iPhone 14 Pro Max, Apple Inc., Cupertino, CA, USA) using the ultra-wide camera (13 mm equivalent focal length) with fixed exposure settings throughout (f/2.2, 1/240 s, ISO 40, 0 EV) and automatic white balance, and were saved as JPEG. Images were subsequently cropped to a common 1:1 frame and lightened using a single preset applied uniformly to all images (Lightroom, Adobe Inc., San Jose, CA, USA), so that transparent and gel-like specimens would be discernible against the white background; no image was adjusted individually, and no selective or regional editing was performed. The 8 mm × 8 mm square mold aperture appears in every frame and provides an internal dimensional reference. Because white balance was automatic and a global tonal adjustment was applied during figure preparation, the photographs were used to assess structural integrity, surface texture, and specimen handling behavior, not for colorimetric comparison between groups. Specimens were photographed both undisturbed within the mold and during an attempt to lift them intact with tweezers, because handling behavior distinguishes a rigid polymer from a gel that can appear superficially cured.

Each specimen was then assigned to one of three mutually exclusive polymerization outcome categories: (1) fully polymerized: a rigid, self-supporting specimen that could be lifted, handled, and clamped intact, retaining its shape and mold dimensions without deformation, and does not imply complete monomer conversion; (2) partially polymerized: a specimen with a hardened but friable, gel-like body (“hard jelly-like”) that deformed or fractured on handling or under the Attenuated Total Reflectance (ATR) clamp; and (3) unpolymerized: a specimen that remained liquid or jelly-like, could not be lifted intact, and flowed or was displaced when the Attenuated Total Reflectance (ATR) crystal clamp was tightened.

One operator prepared and handled specimens, while a second operator captured the photographs. The operator who prepared and handled the specimens classified them based on both the standardized photographs and direct handling at the time of fabrication; the same unblinded operator then performed the DC measurements. The categories were defined by physical handling behavior rather than graded visual judgment; however, because a single operator performed the classification and was aware of group allocation, assessor bias cannot be excluded, and inter-examiner agreement was not assessed. These categories are reported alongside the quantitative DC data.

### 2.5. Degree of Conversion (DC)

The DC was evaluated for each group using Fourier Transform Infrared (FTIR) spectroscopy (Nicolet 6700 FT-IR spectrometer, Thermo Electron Corporation, Waltham, MA, USA) equipped with a diamond ATR crystal (Smart Golden Gate accessory with KRS-5 lenses, Thermo Electron Corporation, Waltham, MA, USA). The ATR diamond crystal area was 2 mm × 2 mm square. The absorbance was measured using 32 scans at 2 cm^−1^ resolution over the range of 4000–400 cm^−1^. Background spectra were recorded before each group.

Specimens were fabricated in a standardized square plastic mold (8 mm × 8 mm; thickness 0.8 mm) resting on a standardized white benchtop surface, with a Mylar strip beneath the specimen. Each specimen was photocured for a standardized 20 s at a fixed distance of 2 mm between the curing tip and the specimen surface using either the SLED or the MLED, giving 96 specimens in total (n = 3 per formulation per LCU).

The specimen, within its mold, was placed on the diamond crystal and compressed with the ATR accessory’s pressure clamp to ensure optical contact. The bottom, Mylar-covered surface faced the crystal, so measurements were taken from the surface opposite the one irradiated, which represents the least-cured region of the increment. We took five measurements from each specimen at standardized locations (the four corners and the center) and used the mean for analysis. Spectra were acquired immediately after photoactivation. When a specimen was only partially polymerized, fewer than five measurements could be obtained; when it had not polymerized, no measurement was possible. In partially polymerized specimens, one to three of the five locations yielded a measurable spectrum, depending on whether an intact, undistorted surface was available at each location, and the specimen mean was calculated from the available locations. The uncured reference ratio was obtained from the corresponding unpolymerized formulation of identical composition, measured under the same conditions immediately before photoactivation. DC was calculated from the reduction of the aliphatic C=C absorption at 1637 cm^−1^ relative to the aromatic C=C reference at 1607 cm^−1^:

(1) $$\text{DC}\;(\%)=[1-\frac{\text{cured}\;(\text{peak height under}\;1637/\text{peak height under}\;1607)}{\text{uncured}\;(\text{peak height under}\;1637/\text{peak height under}\;1607)}]\times 100$$

### 2.6. Statistical Analysis

The DC was analyzed by two-way analysis of variance (ANOVA) with curing unit (MLED, SLED) and formulation (16 groups) as fixed factors, including their interaction. Because the interaction was significant, post hoc comparisons among formulations were made within each LCU using Tukey’s honestly significant difference (HSD) test. Comparisons between the two LCUs within each formulation were made using pairwise comparisons of the estimated marginal means based on the pooled error term of the two-way model, with Bonferroni adjustment for the 16 between-unit comparisons. Statistical significance was set at α = 0.05. Analyses were performed in SPSS Statistics for Windows (version 29; IBM Corp., Armonk, NY, USA).

Specimens classified as unpolymerized could not yield a stable ATR-FTIR spectrum because they lacked structural integrity, so they were entered as 0% DC. This value is an operational convention, not a measurement. Conversion in these specimens was not zero but indeterminate, because too little or no polymer had formed to permit measurement. We adopted this convention so complete polymerization failure would remain visible in the model rather than being deleted as missing data, but it creates groups with zero within-group variance. Model fit statistics and tests of variance homogeneity should therefore be interpreted with that in mind, and for these groups the photographic classification, rather than the imputed zero, is the primary outcome. We assessed homogeneity of variance with Levene’s test. Because the imputed zeros are not measurements, a sensitivity analysis excluding every specimen assigned 0% DC was also performed: (i) a two-way ANOVA restricted to the nine formulations that yielded measurable spectra with both LCUs (the four CQ+EDAB formulations, CQ 0.5 + PPD 0.5, and the four CQ+BAPO formulations), and (ii) one-way ANOVA with Tukey HSD tests within each LCU restricted to the measured groups (13 groups for the MLED and 9 for the SLED). For the same reason, model fit statistics (R^2^) and effect sizes (partial η^2^) from the full model are not reported.

## 3. Results

### 3.1. Polymerization Outcome: Photographic Classification

**Figure 4:**
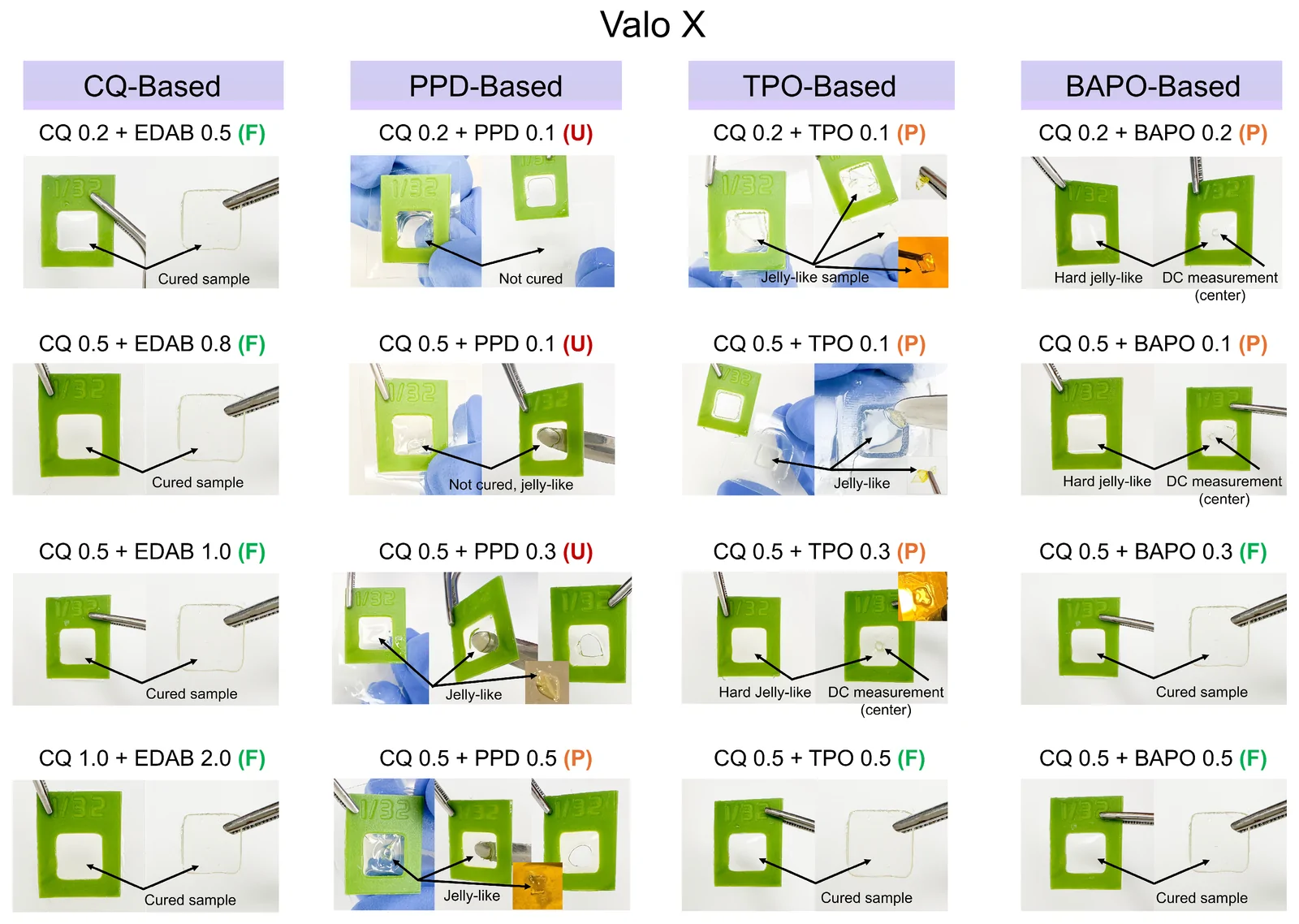
Standardized photographs of every unfilled resin formulation after light-curing with the MLED (Valo X) unit. Specimens are shown within the mold and during an attempt to lift them intact. CQ+EDAB formulations, CQ 0.5 + TPO 0.5, and CQ 0.5 + BAPO 0.3 and 0.5 produced rigid, self-supporting specimens, and do not imply complete monomer conversion (F); the remaining formulations produced partially polymerized (hard, gel-like) (P) or unpolymerized (jelly-like) (U) specimens. Abbreviations: F, fully polymerized specimen; P, partially polymerized specimen; U, unpolymerized specimen.

**Figure 5:**
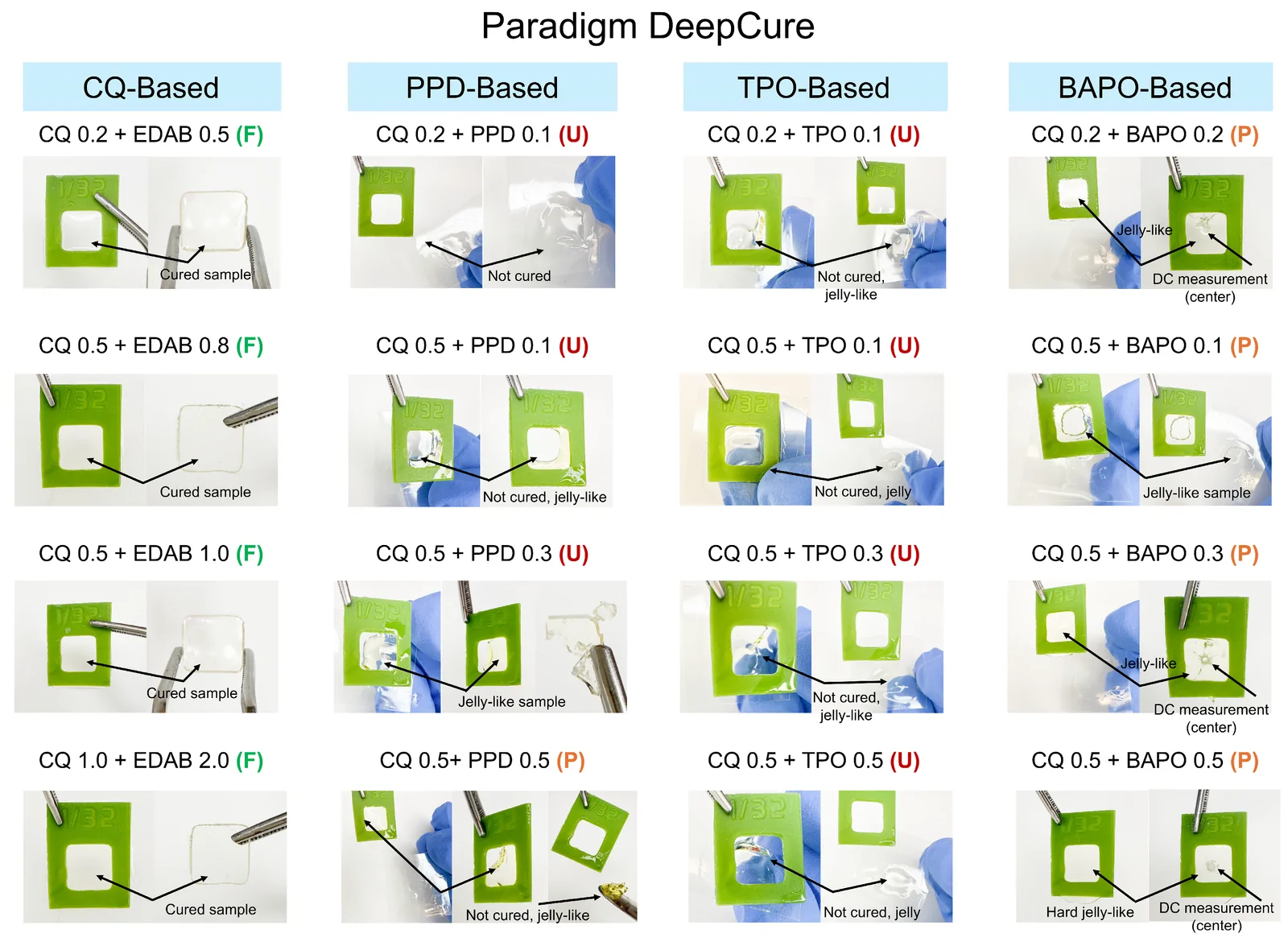
Standardized photographs of the same formulations after light-curing with the SLED (Paradigm DeepCure) unit, captured under identical conditions to Figure 4. Only the CQ+EDAB formulations produced rigid, self-supporting specimens, and this does not imply complete monomer conversion (F). No TPO-containing formulation polymerized at any concentration (U); PPD-containing formulations were unpolymerized (U) or, at the highest concentration, only partially polymerized (P); and all BAPO formulations were partially polymerized (P) at best. Abbreviations: F, fully polymerized specimen; P, partially polymerized specimen; U, unpolymerized specimen.

Figure 4 and Figure 5 show every formulation after curing with the MLED and the SLED, respectively, photographed under identical conditions. The contrast between the two figures is evident without reference to any numerical value. Under the MLED (Figure 4), all four CQ+EDAB formulations, CQ 0.5 + TPO 0.5, and CQ 0.5 + BAPO 0.3 and 0.5, produced rigid, self-supporting specimens that could be lifted intact. Under the SLED (Figure 5), only the four CQ+EDAB formulations did so: no PPD- or TPO-containing formulation produced a specimen that could be lifted intact with that unit at any concentration tested.

The intermediate and failed categories were equally informative. Under the MLED, CQ 0.5 + PPD 0.5, the three lower TPO concentrations, CQ 0.2 + BAPO 0.2, and CQ 0.5 + BAPO 0.1 produced hardened but friable specimens. Under the SLED, CQ 0.5 + PPD 0.5 and all four BAPO formulations were partially polymerized, including CQ 0.5 + BAPO 0.5, which remained hard gel-like on handling despite subsequently yielding a mean DC of 48.1%.

Across the full design, 11 of the 32 groups were classified as fully polymerized, 11 as partially polymerized, and 10 as unpolymerized. The 10 unpolymerized groups, the three lowest PPD formulations under both curing units, and all four TPO formulations under the SLED yielded specimens from which no ATR-FTIR measurement could be obtained at all.

The photographic classifications corresponded to specimen handling characteristics. Fully polymerized specimens were rigid and self-supporting and retained their shape during handling; partially polymerized specimens were hardened but friable or gel-like and deformed or fractured during handling; and unpolymerized specimens remained liquid or jelly-like and could not be lifted intact. Unpolymerized specimens did not provide sufficient structural integrity for ATR-FTIR measurement. The polymerization outcome and mean DC values for each formulation are reported together in Table 2.

**Figure 6:**
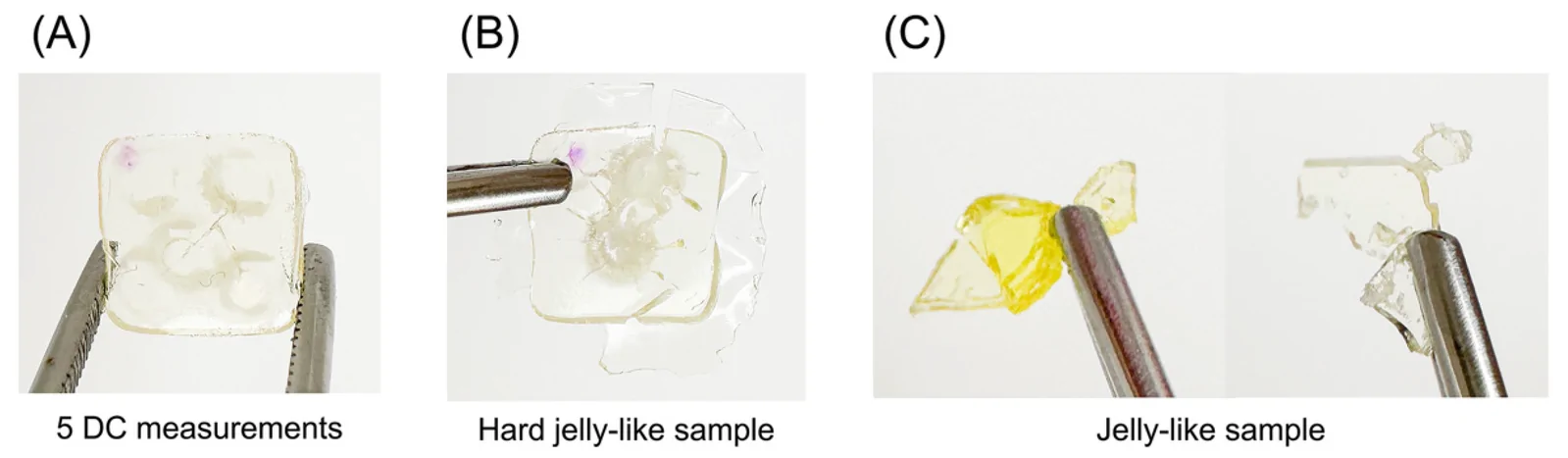
Relationship between polymerization outcome category and the number of ATR-FTIR measurements obtainable. (**A**) A fully polymerized specimen permitted five measurements at the predefined locations (four corners and one center). (**B**) A partially polymerized, hard jelly-like specimen fractured under the ATR clamp, so fewer than five measurements could be obtained. (**C**) An unpolymerized (liquid or jelly-like) specimen flowed or was displaced when the clamp was tightened, so no measurement could be obtained.

Figure 6 shows why the photographic categories governed how much quantitative data each specimen could provide. From a fully polymerized specimen, five measurements were obtained at the predefined locations (Figure 6A). From a partially polymerized, hard jelly-like specimen, the material fractured under the clamp and fewer than five locations could be reached (Figure 6B). From an unpolymerized (liquid or jelly-like) specimen, the material flowed or was displaced when the ATR swivel was tightened, and no measurement was possible (Figure 6C). Formulations described as unpolymerized or jelly-like are therefore those for which no stable ATR-FTIR spectrum could be acquired, and are entered in the analysis as 0% DC under the convention described in Section 2.6.

### 3.2. Degree of Conversion (DC)

**Figure 7:**
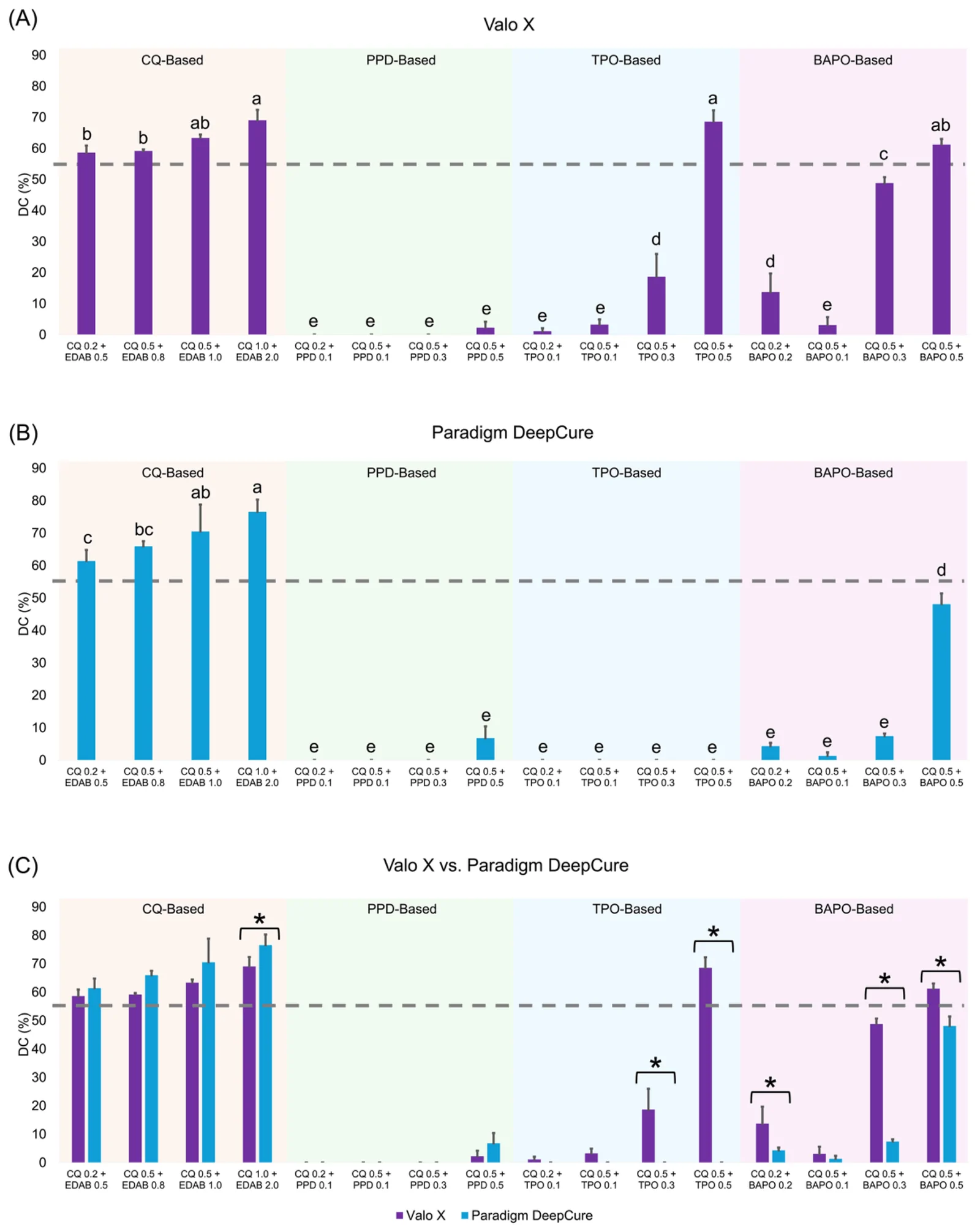
Statistical comparisons of the DC% among groups light-cured with both LCUs. (**A**) Statistical comparisons among groups light-cured with the MLED (Valo X) LCU. (**B**) Statistical comparisons among groups light-cured with the SLED (Paradigm DeepCure) LCU. In (**A**,**B**), groups sharing different letters differ significantly among photoinitiator groups. (**C**) Statistical comparisons between light-curing with the Valo X unit and Paradigm DeepCure LCU for each group. Asterisks indicate significant differences between LCUs within the same formulation *p* < 0.05). Bars show means and error bars show standard deviations (n = 3 specimens per group). The dashed line marks 55% DC, shown only as a reference value.

Mean DC values for every formulation under both curing units are given in Table 2. Because a significant formulation × LCU interaction was present, values are reported separately for each unit rather than pooled. CQ+EDAB was the only system to polymerize fully under both units and produced the highest conversion under the SLED; under the MLED, CQ 0.5 + TPO 0.5 (68.5 ± 3.7%) and CQ 0.5 + BAPO 0.5 (61.2 ± 1.9%) were statistically indistinguishable from CQ 1.0 + EDAB 2.0 (Figure 7A). DC increased with initiator concentration, from 58.6 ± 2.3% (MLED) and 61.3 ± 3.4% (SLED) at CQ 0.2 + EDAB 0.5 to 69.0 ± 3.4% (MLED) and 76.5 ± 3.8% (SLED) at CQ 1.0 + EDAB 2.0. Mean DC was numerically higher with the blue-only SLED than with the MLED in every CQ+EDAB formulation, but the difference was statistically significant only for CQ 1.0 + EDAB 2.0 (Figure 7C). The dotted line in Figure 7 marks 55% conversion, a value that has been associated with acceptable clinical performance in some previous work [35] and that is used here only as a reference point.

The CQ+PPD formulations, which contained no amine co-initiator, failed under both LCUs. Three of the four formulations produced specimens from which no measurement could be obtained, and the highest concentration reached only 2.2 ± 1.9% (MLED) and 6.7 ± 3.6% (SLED).

CQ+TPO produced the clearest dissociation between the two units. Under the MLED, DC increased with TPO concentration: 1.1 ± 0.9%, 3.2 ± 1.7%, 18.6 ± 7.3%, and 68.5 ± 3.7% at CQ 0.5 + TPO 0.5, a value not significantly different from that of the best CQ+EDAB formulation (CQ 1.0 + EDAB 2.0, 69.0 ± 3.4%; Figure 7A). Under the SLED, no TPO formulation at any concentration produced a measurable specimen; a fivefold increase in TPO content did not alter the outcome.

CQ+BAPO responded to both LCUs but far more strongly to the MLED and was the only alternative system to reach substantial conversion under the blue-only unit. At CQ 0.5 + BAPO 0.3, DC was 48.8 ± 2.0% (MLED) versus 7.4 ± 0.7% (SLED); at CQ 0.5 + BAPO 0.5 it was 61.2 ± 1.9% versus 48.1 ± 3.3%. The difference between units for CQ 0.5 + BAPO 0.5 concentration, therefore, narrowed as BAPO concentration increased.

Figure 7 shows the statistical comparisons among formulations for each curing unit (Figure 7A,B) and between the two units within each formulation (Figure 7C). The balanced analysis included all 96 specimens across 16 formulations and two curing units (n = 3/subgroup). Model fit statistics are not reported because they are dominated by the groups assigned a constant value of 0% (Section 2.6).

**Figure 8:**
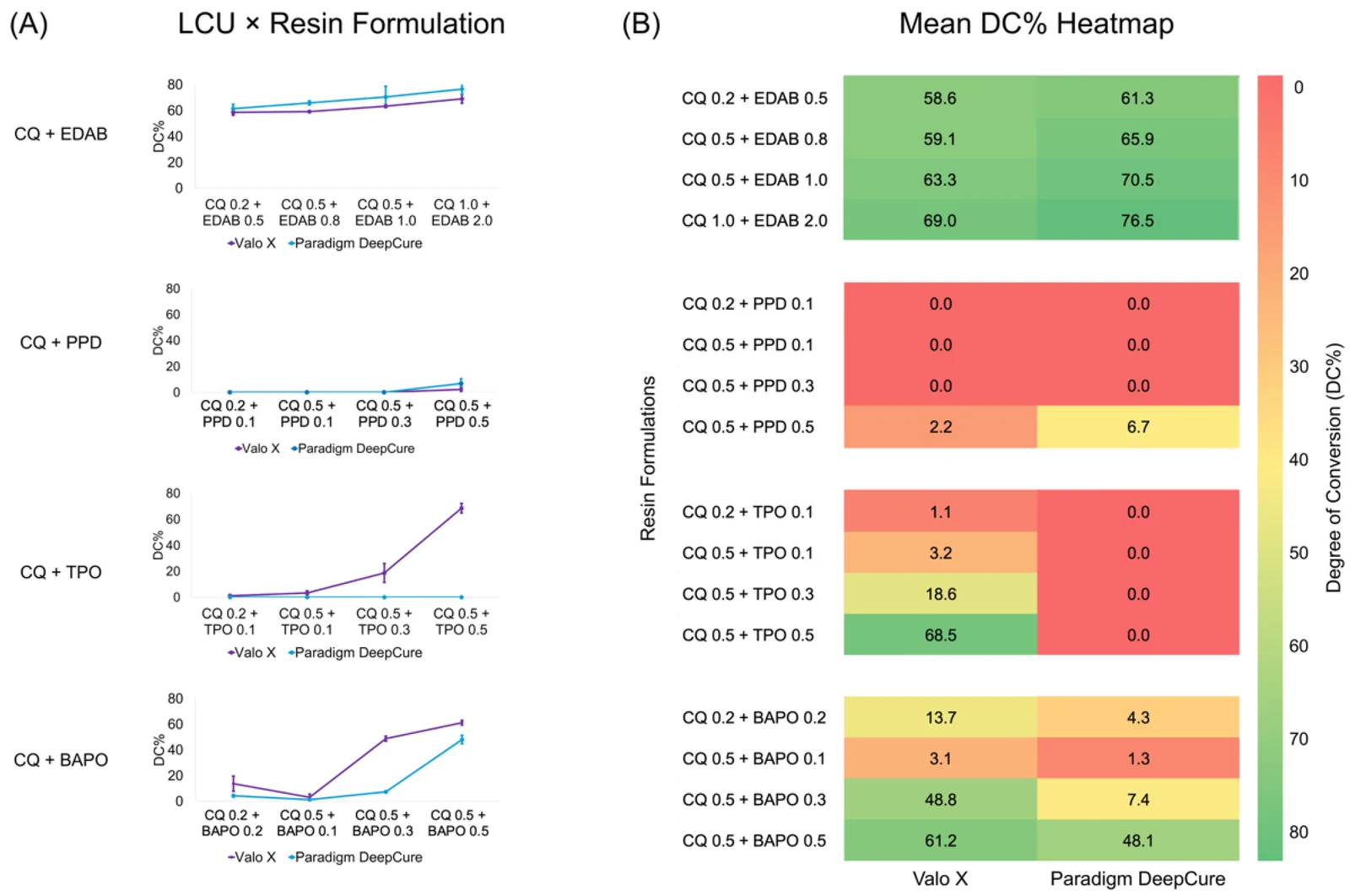
Effect of LCU and photoinitiator formulation on the DC. (**A**) Interaction plot showing mean DC values and standard deviation (SD) for each formulation polymerized using the Valo X and Paradigm DeepCure LCUs. (**B**) Heatmap representation of mean DC values across formulations and LCUs. Green color indicates higher DC values, while yellow-orange-red colors indicate reduced polymerization efficiency.

A significant main effect of LCU was observed on DC (F = 186.564, *p* < 0.001), indicating that polymerization efficiency differed between the Paradigm DeepCure and Valo X LCUs. Formulation also had a highly significant effect on DC (F = 583.790, *p* < 0.001). In addition, a significant LCU × formulation interaction was detected (F = 74.542, *p* < 0.001), indicating that the effect of the LCU depends on the photoinitiator system tested. As illustrated in Figure 8A, the interaction plot showed distinct response patterns among formulations, with several systems exhibiting substantial divergence between LCUs. In particular, TPO-based systems demonstrated the greatest separation between curing units, whereas CQ+EDAB formulations maintained consistently high DC values across both LCUs. Figure 8B further highlights the overall polymerization landscape via a heatmap, clearly distinguishing high-performing CQ+EDAB systems from poorly polymerized PPD-based formulations.

Tukey post hoc analysis identified five statistically distinct homogeneous subsets among formulations. The CQ+EDAB formulations consistently clustered within the highest-performing subsets, whereas PPD-based systems clustered within the lowest-performing groups.

Levene’s test based on means indicated heterogeneity of variance among groups (*p* < 0.001), whereas the median-based statistic was not significant (*p* = 0.146). The mean-based heterogeneity is an expected consequence of the groups with zero within-group variance created by the imputation convention described in Section 2.6 rather than of unstable measurement, and results were interpreted accordingly.

Excluding every specimen assigned 0% DC did not change the principal findings. In the two-way ANOVA restricted to the nine formulations measurable with both LCUs, formulation (F(8,36) = 469.7), LCU (F(1,36) = 21.3), and their interaction (F(8,36) = 35.1) all remained significant (*p* < 0.001). Between-unit differences remained significant for CQ 0.2 + BAPO 0.2, CQ 0.5 + BAPO 0.3, and CQ 0.5 + BAPO 0.5, whereas the 7.5-percentage-point difference for CQ 1.0 + EDAB 2.0 was significant before (*p* = 0.008) but not after Bonferroni adjustment for nine comparisons (*p* = 0.072). In the Tukey analyses within each LCU restricted to measured groups, 76 of 78 pairwise decisions among the 13 MLED groups and 35 of 36 among the 9 SLED groups were unchanged relative to the same analyses including the imputed values. The two changes both concerned high-conversion groups (MLED: CQ 0.5 + EDAB 0.8 vs. CQ 0.5 + TPO 0.5, *p* = 0.034 vs. 0.068; SLED: CQ 0.2 + EDAB 0.5 vs. CQ 0.5 + EDAB 1.0, *p* = 0.020 vs. 0.116), and CQ 0.5 + TPO 0.5 remained statistically indistinguishable from CQ 1.0 + EDAB 2.0 under the MLED in both analyses (*p* > 0.99).

### 3.3. Concordance Between Photographic and Spectroscopic Outcomes

The two outcomes were ordered consistently across all 32 groups. Every group classified photographically as fully polymerized yielded a mean DC of 48.8% or higher; every group classified as partially polymerized yielded a value below that threshold; and every group classified as unpolymerized yielded no measurable value at all. In the 10 unpolymerized groups, the photographic classification was the only recorded outcome.

## 4. Discussion

Polymerization of these model resins depended first on whether the curing unit emitted photons the photoinitiator could absorb; only when that condition was met did the amount of light delivered influence the outcome. The most direct demonstration is the CQ+TPO series. Under the MLED, conversion increased with TPO concentration to 68.5%, whereas under the SLED, which delivered approximately 32% higher irradiance and 33% higher radiant exposure than the MLED but emitted no violet light, not one formulation at any concentration produced a measurable or handleable specimen. The converse case is equally informative. For CQ+EDAB, both units emit within the CQ absorption band, and there the higher-output SLED produced numerically higher conversion in every formulation, significantly so only at the highest initiator concentration. Within the absorption band, conversion therefore depended on irradiance, radiant exposure, and initiator concentration; outside that band, additional radiant exposure produced no usable polymer. The alternative hypotheses were therefore only partially supported. The first was supported for the SLED, with which only CQ+EDAB produced fully polymerized specimens, but not for the MLED, with which CQ 0.5 + TPO 0.5 and CQ 0.5 + BAPO 0.3 and 0.5 were also fully polymerized. The second was supported for the SLED and with respect to the interaction between the photoinitiator system and LCU, but not for the MLED, with which CQ 0.5 + TPO 0.5 and CQ 0.5 + BAPO 0.5 were statistically indistinguishable in DC from the best CQ+EDAB formulation.

The specimens were light-cured at a fixed, clinically relevant distance of 2 mm, at which beam divergence provided coverage of the 8 mm × 8 mm specimens (11.3 mm diagonal) despite the 10-mm tip diameter of the Paradigm DeepCure. Nevertheless, the beam profiles of the two LCUs differed substantially (Figure 3). The Valo X exhibited a comparatively broad and homogeneous irradiance distribution across its 12-mm tip, whereas the Paradigm DeepCure showed a centrally concentrated high-irradiance region with markedly lower irradiance toward the periphery. Consequently, the relatively high measurable DC of CQ 0.5 + BAPO 0.5 under the SLED despite its gel-like handling characteristics may reflect spatially nonuniform polymerization, whereby regions receiving greater irradiance achieved measurable conversion while less-irradiated peripheral regions remained inadequately polymerized. ATR sampling was selected because it allowed direct, non-destructive measurement of the intact 0.8 mm specimens within the mold. Because the evanescent wave penetrates only a few micrometers into the specimen, each spectrum characterized the surface in contact with the crystal, which in this study was the least-irradiated (bottom) surface.

The photographic record is what makes the effect of spectral mismatch visible rather than merely inferable, and it is the principal methodological contribution of this study. Conventional reporting of these experiments is structurally biased toward success. A specimen that does not polymerize cannot be clamped in an ATR accessory, cannot yield a spectrum, and therefore leaves the dataset either as missing data or as a zero that reads like a measurement rather than like a failure. Photographing every specimen, including those that could not be measured, converts that absence into evidence. Figure 4 and Figure 5 present the same 16 formulations under the two curing units, and the difference in polymerization outcomes is legible at a glance. Where some specimens in some formulations can be lifted intact with tweezers under one unit, they remain liquid or gel-like under the other. Importantly, the term “fully polymerized” does not imply complete monomer conversion. Rather, it refers to specimens that maintained their structural integrity during handling, allowing five measurements to be obtained without specimen deformation, fracture, or destruction.

Figure 6 makes the mechanism of data loss explicit, showing why five, fewer than five, or no ATR measurements were obtainable from specimens of decreasing structural integrity. Photographic and spectroscopic outcomes were ordered consistently in every group, but the photographs carried information in exactly those groups where spectroscopy was impossible, which are the groups of greatest clinical concern.

A further advantage of the visual record emerged where the two outcomes diverged in emphasis. CQ 0.5 + BAPO 0.5 cured with the SLED produced a mean DC of 48.1%, a value that in isolation might be reported as moderate conversion, yet the specimen could not be handled without deforming. The most likely explanation is the nonuniform exposure, which resulted in the corners receiving considerably less light than the center.

CQ+EDAB behaved as expected of a spectrally matched Type II system. Upon absorbing blue light, CQ reaches an excited triplet state and forms a transient exciplex with the tertiary amine, from which electron transfer and subsequent proton transfer generate the free radical that initiates propagation; without an amine, CQ initiates poorly [26,36,37]. Conversion increased with initiator concentration across the series, and the highest value recorded in the study, 76.5%, was obtained from CQ 1.0 + EDAB 2.0 under the SLED. That the blue-only unit produced numerically higher conversion for this system, significantly so only at the highest concentration, is not paradoxical. The SLED concentrated its higher radiant exposure entirely within the CQ absorption band, whereas the MLED distributed a lower total output across a wider range, part of which CQ absorbs only weakly. This comparison serves as the positive control for the study’s central interpretation: where spectral overlap is adequate, conversion follows the delivered energy and initiator concentration.

The PPD groups failed under both curing units, for two reasons acting together. Its absorption maximum near 410 nm lies at the margin of the MLED violet emission and entirely outside the SLED output, and its molar absorptivity in this region is low. In addition, EDAB was deliberately omitted from the CQ+PPD, CQ+TPO, and CQ+BAPO formulations so that the contribution of each alternative initiator could be isolated. PPD, like CQ, is a Type II initiator that requires an amine to generate radicals efficiently, so its failure here reflects the combined absence of a co-initiator and of adequate spectral overlap. These data therefore establish that PPD cannot compensate for a missing amine even at elevated concentration, but they do not represent the performance of PPD in an optimized commercial formulation containing one and should not be read as such.

The CQ+TPO system showed dependence on the curing unit and, taken together with the photographs, provides the clearest evidence that adequate spectral overlap is a prerequisite for polymerization; where that prerequisite was met, the response was graded by initiator concentration. As a Type I initiator, TPO cleaves directly on absorption and needs no co-initiator, but its absorption peaks at about 380 nm and ends near 420 nm. The MLED violet emitter supplies that band; the SLED does not. Under the MLED, the system showed a clear concentration–response relationship, conversion rising with concentration to a value comparable with the best CQ+EDAB formulations. Under the SLED, all four formulations produced the same result at every concentration: no usable polymer. Increasing TPO content fivefold did not move the outcome, because the limiting factor was not the number of initiator molecules but the absence of photons they could absorb. This is the practical expression of the Grotthuss–Draper and Stark–Einstein laws in a material a clinician might hold.

Spectral overlap is nevertheless a necessary rather than a sufficient condition for efficient initiation. The number of photons a photoinitiator absorbs depends on its molar absorptivity at each emitted wavelength and on its concentration, and the photon absorption efficiency of a photoinitiator–LCU pair can be estimated from the overlap of the photoinitiator extinction spectrum with the LCU spectral irradiance [12]. How effectively the absorbed photons initiate polymerization then depends on the quantum yield of radical formation and on the reactivity of the radicals produced [19]. CQ absorbs weakly (≈46 L·mol^−1^·cm^−1^ at 469 nm), is consumed with a low quantum yield (approximately 0.07 per absorbed photon), and relies on bimolecular electron and hydrogen transfer from the amine to form the initiating aminoalkyl radical, whereas the acylphosphine oxides absorb more strongly in the violet and undergo unimolecular α-cleavage to benzoyl and phosphinoyl radicals, the latter being highly reactive toward methacrylate double bonds [18,26]. These differences help explain why CQ 0.5 + TPO 0.5 reached conversion comparable with that of the best CQ+EDAB formulation under the MLED, although only part of the MLED output lies in the violet band, and why BAPO, which carries two cleavable acyl groups and absorbs further toward the blue, retained partial activity under the SLED. A quantitative analysis of photon absorption and radical yield for these formulations, which would require molar-normalized absorption spectra measured in the resin, was beyond the scope of this study.

The BAPO showed an intermediate position consistent with its broader absorption. Like TPO, it is a Type I initiator, but its absorption extends further toward the blue, so a portion of the SLED output remains usable. The data reflect that BAPO was the only alternative initiator to reach substantial conversion under the blue-only unit (48.1% at the highest concentration), while still performing markedly better under the MLED (61.2%), and the difference in DC between units decreased as the BAPO concentration increased (CQ 0.5 + BAPO 0.5), which suggests equal concentration ratios provide the best conversion. Concentration alone was nonetheless insufficient at lower loadings, where conversion under the SLED remained below 8%, and as noted above, even the highest-conversion SLED specimen was structurally inadequate, an outcome to which the nonuniform SLED beam probably contributed. Resin composition, light transmission, and spectral matching therefore remain jointly determining [12,13,26,27,38].

Because the absorbance values in Figure 2A are not normalized to molar concentration, they show the wavelength range over which each photoinitiator absorbs but not its relative absorption efficiency. The photoinitiators differ markedly in molar absorptivity [12]. CQ absorbs weakly at its blue maximum (molar extinction coefficient ≈ 46 L·mol^−1^·cm^−1^ at 469 nm) [19] whereas the acylphosphine oxides absorb considerably more strongly in the violet region, so the number of photons absorbed by a formulation depends on the product of the LCU spectral irradiance, the molar absorptivity of the photoinitiator at each wavelength, and its concentration [18]. EDAB is not shown in Figure 2A because it absorbs only in the ultraviolet (maximum ≈ 310 nm), outside the emission range of both LCUs; it acts as an electron and hydrogen donor to excited CQ and does not itself need to absorb light [39]. Likewise, the Bis-GMA/TEGDMA monomers are colorless and do not absorb appreciably at the 385–515 nm wavelengths emitted by the LCUs, so they did not compete with the photoinitiators for the emitted photons [40].

For esthetic restorative practice, the implication is specific. Alternative photoinitiators exist because CQ is yellow, and the materials in which they are used, high-translucency enamel shades, bleach shades, single-shade universal RBCs, and light-cured esthetic cements, are precisely those in which the clinician has least visual and tactile feedback about whether curing has succeeded. A thin, translucent, highly esthetic increment that has not polymerized does not always announce itself. The consequences are well documented: insufficient activation reduces conversion and increases the release of residual monomer into the oral environment [1,2,3,41]. Within the limits of a model-resin study using two LCUs, the present results suggest that a curing unit should not be selected on irradiance alone, and that a high-output, blue-only LED may be poorly suited to a material that depends on violet-absorbing photoinitiators. This argues for routine disclosure of photoinitiator composition and required spectral output by manufacturers, and for clinicians to confirm that their curing unit emits violet light before placing materials that depend on it.

Several limitations qualify these findings. Unfilled model resins were used deliberately to isolate initiator chemistry; commercial RBCs contain fillers, pigments, opacifiers, stabilizers, and proprietary initiator blends that alter light transmission and scattering, so these results provide mechanistic insight rather than a prediction of clinical performance, and the absolute DC values should not be transferred to filled materials. Although beam divergence at the 2-mm curing distance provided coverage of the 8 mm × 8 mm specimen with the 10-mm SLED tip, its beam profile was markedly nonuniform, with substantially lower irradiance toward the periphery, whereas the 12-mm MLED exhibited a comparatively homogeneous beam profile (Figure 3). Consequently, the SLED DC and handling outcomes may reflect spatially variable exposure, particularly toward the specimen periphery, and between-unit comparisons reflect differences in both emission spectrum and spatial irradiance distribution. EDAB was omitted from the alternative-initiator formulations, which isolates each initiator but does not reflect commercial practice. Three specimens per group is a small sample, adequate for the large and largely categorical effects observed here but insufficient to resolve small differences. Specimens that could not be measured were entered as 0% DC, an operational convention that inflates model fit and produces the mean-based variance heterogeneity detected by Levene’s test, although a sensitivity analysis excluding these values did not change the principal findings. Only two curing units, one exposure time, one specimen thickness, and one tip distance were evaluated, and properties beyond DC, depth of cure, shrinkage stress, color stability, and monomer elution were not measured. Finally, photographic classification was performed by a single operator with knowledge of group allocation, so assessor bias cannot be excluded; blinded, independently scored assessment with reported inter-examiner agreement would strengthen future studies of this design.

Future work should extend the photographic approach to model-filled resin systems of greater optical complexity, in which light transmission and scattering interact with spectral compatibility, should use exposure conditions in which the curing tip fully covers the specimen, with similar beam profiles, and should pair these with measurements of bacterial growth, fibroblast cytotoxicity, and monomer elution from the failed and partially polymerized specimens that this design is able to preserve and characterize.

## 5. Conclusions

Based on the investigated unfilled model resins and two LED curing units, it can be concluded that (1) photographically classified polymerization outcomes and degree of conversion differed among the tested photoinitiator formulations and between the single-emission-peak and multiple-emission-peak LED curing lights; (2) the camphorquinone/amine system polymerized with both curing units, whereas the phosphine oxide initiators required violet emission to polymerize fully, and 1-phenyl-1,2-propanedione formulated without an amine co-initiator did not polymerize adequately with either unit, a result that cannot be generalized to amine-containing formulations; and (3) photographic documentation recorded polymerization failure where degree of conversion could not be measured and is a useful complement to spectroscopic analysis. These findings suggest that restorative materials containing alternative photoinitiators should not be assumed to cure adequately with every LED curing unit and that spectral compatibility should be verified rather than presumed; confirmation with filled commercial materials and a wider range of curing units is required before clinical recommendations can be made.

## Figures and Tables

**Table 1:** Composition and manufacturers of the resin matrix components and photoinitiators used in the study.

Component	Material	Abbreviation	Manufacturer	Lot No.
Resin Matrix				
Monomer	Bisphenol A glycerolate dimethacrylate, Triethylene glycol dimethacrylate solution (1:1 premixed resin blend)	Bis-GMA, TEGDMA	Esstech, Inc. Essington, PA, USA	818–497
Photoinitiators				
Norrish Type II or Diketone	Camphorquinone (97%)	CQ	Sigma-Aldrich, St. Louis, MO, USA	102733174
	1-phenyl-1,2-propanedione	PPD	Sigma-Aldrich, St. Louis, MO, USA	1003440013
Tertiary amine (co-initiator)	Ethyl 4-(dimethylamino)-benzoate	EDAB	Esstech, Inc. Essington, PA, USA	23LA5455
Norrish Type I	Diphenyl(2,4,6-trimethylbenzoyl)phosphine oxide	TPO	Sigma-Aldrich, St. Louis, MO, USA	1003620159
	Phenylbis(2,4,6-trimethylbenzoyl)phosphine oxide	BAPO	Sigma-Aldrich, St. Louis, MO, USA	1003716569

Abbreviations: CQ, camphorquinone; EDAB, ethyl 4-(dimethylamino)-benzoate; PPD, 1-phenyl-1,2-propanedione; TPO, diphenyl(2,4,6-trimethylbenzoyl)phosphine oxide; BAPO, phenylbis(2,4,6-trimethylbenzoyl)phosphine oxide.

**Table 2:** Polymerization outcome and mean degree of conversion (DC%) with standard deviations (SD) for all formulations cured with each light-curing unit.

Photoinitiator Type	Photoinitiator Concentration Ratio Group (wt%)	Valo X Mean (SD)	Valo X Photo Outcome	Paradigm DeepCure Mean (SD)	Paradigm DeepCure Photo Outcome
CQ-based	CQ 0.2 + EDAB 0.5	58.6 (2.3)	F	61.3 (3.4)	F
	CQ 0.5 + EDAB 0.8	59.1 (0.4)	F	65.9 (1.6)	F
	CQ 0.5 + EDAB 1.0	63.3 (1.1)	F	70.5 (8.2)	F
	CQ 1.0 + EDAB 2.0	69.0 (3.4)	F	76.5 (3.8)	F
PPD-based	CQ 0.2 + PPD 0.1	0 *	U	0 *	U
	CQ 0.5 + PPD 0.1	0 *	U	0 *	U
	CQ 0.5 + PPD 0.3	0 *	U	0 *	U
	CQ 0.5 + PPD 0.5	2.2 (1.9)	P	6.7 (3.6)	P
TPO-based	CQ 0.2 + TPO 0.1	1.1 (0.9)	P	0 *	U
	CQ 0.5 + TPO 0.1	3.2 (1.7)	P	0 *	U
	CQ 0.5 + TPO 0.3	18.6 (7.3)	P	0 *	U
	CQ 0.5 + TPO 0.5	68.5 (3.7)	F	0 *	U
BAPO-based	CQ 0.2 + BAPO 0.2	13.7 (5.9)	P	4.3 (1.0)	P
	CQ 0.5 + BAPO 0.1	3.1 (2.5)	P	1.3 (1.1)	P
	CQ 0.5 + BAPO 0.3	48.8 (2.0)	F	7.4 (0.7)	P
	CQ 0.5 + BAPO 0.5	61.2 (1.9)	F	48.1 (3.3)	P

Polymerization outcome was assigned photographically and by handling behavior: Full, a rigid, self-supporting specimen that could be lifted, handled, and clamped intact; Partial, a hardened but friable, gel-like specimen that deformed or fractured on handling or under the ATR clamp; Unpolymerized, a specimen that remained liquid or jelly-like and from which no ATR-FTIR measurement could be obtained. * Formulations recorded as 0 are those classified photographically as unpolymerized; the value is an operational convention and not a measured conversion (Section 2.6). Abbreviations: ATR-FTIR, attenuated total reflectance Fourier transform infrared spectroscopy; BAPO, phenylbis(2,4,6-trimethylbenzoyl)phosphine oxide; CQ, camphorquinone; DC, degree of conversion; EDAB, ethyl 4-(dimethylamino)-benzoate; PPD, 1-phenyl-1,2-propanedione; TPO, diphenyl(2,4,6-trimethylbenzoyl)phosphine oxide; F, fully polymerized specimen; P, partially polymerized specimen; U, unpolymerized specimen.

## Data Availability

The original contributions presented in this study are included in the article. Further inquiries can be directed to the corresponding author.

## Abbreviations

The following abbreviations are used in this manuscript:

ATR-FTIR	Attenuated total reflectance Fourier transform infrared spectroscopy
BAPO	Phenylbis(2,4,6-trimethylbenzoyl)phosphine oxide
Bis-GMA	Bisphenol A glycerolate dimethacrylate
CQ	Camphorquinone
DC	Degree of conversion
EDAB	Ethyl 4-(dimethylamino)benzoate
LCU	Light-curing unit
LED	Light-emitting diode
MLED	Multiple-emission-peak LED
PPD	1-Phenyl-1,2-propanedione
RBC	Resin-based composite
SLED	Single-emission-peak LED
TEGDMA	Triethylene glycol dimethacrylate
TPO	Diphenyl(2,4,6-trimethylbenzoyl)phosphine oxide
